# Aspects épidémiologiques, cliniques et thérapeutiques des tumeurs ovariennes présumées bénignes à l’Hôpital Central de Yaoundé

**DOI:** 10.11604/pamj.2016.25.207.9939

**Published:** 2016-11-30

**Authors:** Jeanne Hortence Fouedjio, Florent Ymele Fouelifack, Nadège Amougou Nnang, Robinson Enow Mbu

**Affiliations:** 1 Unité de Gynécologie et Obstétrique de l’Hôpital Central de Yaoundé Cameroun; 2 Département de Gynécologie et Obstétrique de la Faculté de Médecine et des Sciences Biomédicales de l’Université de Yaoundé I, Yaoundé, Cameroun; 3 Département de Gynécologie et Obstétrique de l’Institut Supérieur de Technologies Médicales de Nkolondom, Yaoundé, Cameroun

**Keywords:** Tumeur, ovaire, bénigne, Tumour, ovary, benign

## Abstract

**Introduction:**

Les tumeurs ovariennes présumées bénignes (TOPB) constituent l'un des motifs les plus fréquents de consultation gynécologique, et l'une des indications les plus fréquentes en chirurgie gynécologique.

**Méthodes:**

Nous avons mené une étude descriptive rétrospective dans le service de gynécologie-obstétrique de l'hôpital central de Yaoundé. La durée de l'étude était de 6 mois sur une période 5ans du 1er janvier 2010 au 31 décembre 2014. La population de l'étude était constituée de toutes les patientes opérées pour une tumeur ovarienne présumée bénigne pendant la période de l'étude.

**Résultats:**

L'âge moyen était de 29,33± 6,83 avec un âge minimum de 20 ans et un âge maximum de 48ans. La tranche d'âge la plus représentée est celle de 21à 25 ans. Le motif de consultation était les douleurs pelviennes 93,90% des cas. Onze patientes avaient une grossesse soit 33,3% de notre population d'étude et 72,70% étaient au premier trimestre de la grossesse. Les TOPB répertoriés dans notre série étaient : la rupture de kyste (36,40%), la torsion d'annexe (27,30%), le volumineux kyste ovarien (21,20%), l'hémorragie intrakystique (15,20%). Le traitement était conservateur chez 26 (78,78%) patientes. Cependant, l'annexectomie a été réalisée chez 5 patientes sur 9 qui ont eu une torsion d'annexe.

**Conclusion:**

Les TOPB compliquées sont présentes dans notre milieu surtout chez des patientes en âge de procréer. Le diagnostic tardif particulièrement en cas de torsion d'annexe oblige un traitement radical qui est délétère pour le patient. Le traitement chirurgical par laparoscopie est le plus recommandé si le plateau technique le permet car il diminue les risques de complications.

## Abstract (en)

**Introduction:**

Supposed benign ovarian tumors (SBOT) are considered one of the most frequent reasons for gynecological consultation and one of the most common indications for gynecological surgery.

**Methods:**

We conducted a retrospective descriptive study in the Department of Obstetrics and Gynecology at the Central Hospital of Yaoundé. We carried out a 6-month study, over a five-year period, from 1 January 2010 to 31 December 2014. The study population consisted of all patients undergoing surgery for the treatment of supposed benign ovarian tumor during the study period.

**Results:**

The mean age was 29.33 ± 6.83 with a minimum age of 20 years and a maximum age of 48 years. The most represented age range was 21-25 years. The reason for consultation was pelvic pain in 93.90% of the cases. Eleven patients were pregnant women (33.3% of our study population) and 72.70% were in their first trimester. SBOTs in our series were: cyst rupture (36.40%), ovarian torsion (adnexal torsion) (27.30%), voluminous ovarian cyst (21.20%) intracystic haemorrhage (15.20% ). 26 (78.78%) patients were treated conservatively. However, adnexectomy was performed in 5 out of 9 patients with ovarian torsion.

**Conclusion:**

Complex SBOTs can occur in our mids, especially in patients of reproductive age. Late diagnosis, especially in the case of ovarian torsion, obliges to perform radical procedure with deleterious effects upon the patient. Laparoscopy is the most recommended surgical procedure if the technical equipment is available because it reduces the risk of complications.

## Introduction

Les tumeurs ovariennes présumées bénignes (TOPB) constituent l´un des motifs les plus fréquents de consultation gynécologique et l´une des indications les plus fréquentes en chirurgie gynécologique. Environ 5% des femmes développent un kyste de l´ovaire au cours de leur vie, ils peuvent survenir à tout âge et sont bénins dans 95% des cas [1]. La prévalence des TOPB est estimée entre 14 et 18% chez les femmes ménopausées et de 7% chez les femmes asymptomatiques en période d'activité génitale. Leur incidence pendant la grossesse est de 0,2 à 5% [2]. Les complications des TOPB sont la torsion, la rupture, la compression mécanique des organes de voisinage, l'hémorragie intra kystique, l'infection subaigüe et parfois pendant la grossesse ils peuvent être responsable de dystocies des parties molles [3]. L'installation d'une complication de TOBP impose un traitement qui est le plus souvent chirurgical. Nous avons mené cette étude donc l'objectif général était d'étudier les aspects épidémiologiques, cliniques et thérapeutiques des tumeurs ovariennes présumées bénignes compliquées chez les patientes prises en charges à l'hôpital central de Yaoundé et plus spécifiquement de décrire les caractéristiques sociodémographiques de ces patientes, décrire la présentation clinique de ces trouvailles, décrire les trouvailles peropératoires, répertorier les gestes opératoires.

## Méthodes

Nous avons mené une étude descriptive rétrospective dans le service de gynécologie-obstétrique de l'hôpital central de Yaoundé. La durée de l'étude était de 6 mois sur une période de 5 ans du 1^er^ janvier 2010 au 31 décembre 2014. La population de l'étude était constituée de toutes les patientes opérées pour une tumeur ovarienne présumée bénigne pendant la période de l'étude et dont le dont le dossier a été exploitable. L'échantillonnage a été consécutif pendant la période de l'étude. A partir des registres des comptes rendus opératoires, nous avons recensé les noms des patientes opérées pour tumeurs ovariennes présumées bénignes compliquées et nous nous sommes rendus aux archives pour prendre les dossiers. La collecte des données s'est faite à l'aide d'une fiche technique conçue pour l'étude. Les variables étudiées étaient : les caractéristiques socio-démographiques (âge, statut matrimonial, profession, niveau d'instruction); les caractéristiques cliniques; Le diagnostic préopératoire; les trouvailles peropératoires; les gestes opératoires posés. L'analyse des données s'est faite grâce au logiciel Epi info, les tableaux réalisés à l'aide de microsoft Excel. Une clairance éthique a été obtenue auprès du comité constitutionnel d'éthique de la Faculté de Médecine de l'Université de Yaoundé I, et une autorisation de recherche obtenue auprès du directeur de l'hôpital central de Yaoundé.

## Résultats

Au total 33 dossiers ont été retenus.

**Caractéristiques sociodémographiques:** le Tableau 1 montre les caractéristiques sociodémographiques de notre population d'étude. L'âge moyen était de 29,33± 6,83 avec un âge minimum de 20 ans et un âge maximum de 48 ans. La tranche d'âge la plus représentée est celle de 21à 25 ans.

**Tableau 1:** Caractéristiques sociodémographiques de notre population d’étude

Caractéristiques	Sous- groupe	effectif	pourcentage
**Age**			
	≤20ans	1	3,10%
	21-25ans	12	37,50%
	26-30ans	8	25,00%
	31-35ans	7	21,90%
	36-40ans	2	6,30%
	41-45ans	2	6,30%
	>45ans	1	3,10%
**Profession**			
	Non précisée	1	3,00%
	Etudiante/élève	14	42,40%
	ménagère	7	21,20%
	Secteur informel	5	15,20%
	Secteur privée	5	15,20%
	Secteur public	1	3,00%
**Statut matrimonial**			
	Mariée	9	27,30%
	Célibataire	24	72,70%
**Niveau d'instruction**			
	primaire	5	15,20%
	secondaire	14	42,40%
	universitaire	14	42,40%

L’âge moyen était de 29,33± 6,83 avec un âge minimum de 20ans et un âge maximum de 48ans. La tranche d’âge la plus représentée est celle de 21à 25 ans.

**Caractéristiques cliniques:** le Tableau 2 montre la répartition des patientes selon les différents motifs de consultation. Le motif de consultation était les douleurs pelviennes 93,90% des cas. Onze patientes avaient une grossesse soit 33,3% de notre population d'étude et 72,70% étaient au premier trimestre de la grossesse.

**Tableau 2:** Répartition des patientes selon le motif de consultation

Motif de consultation		Effectif	Pourcentage
Sensation de masse pelvienne		5	15,20%
Troubles du cycle		3	9,00%
Douleur pelviennes		31	93,90%
Grossesse			
	oui	11	33,30%
	non	22	33,30%
Trimestre de la grossesse			
	T1	8	72,70%
	T2	2	18,20%
	T3	1	9,10%

Le motif de consultation était les douleurs pelviennes 93,90% des cas. Onze patientes avaient une grossesse soit 33,3% de notre population d’étude et 72,70% étaient au premier trimestre de la grossesse.

**Diagnostic préopératoire:** le Tableau 3 montre les différents diagnostics avant la chirurgie Les diagnostics les plus souvent posé initialement étaient les grossesses extra-utérines et les torsions d'annexes.

**Tableau 3:** Distribution des patientes selon le diagnostic préopératoire

Diagnostic préopératoire	Effectif	Pourcentage
Torsion d'annexe	10	30,30%
Rupture de kyste ovarien	1	3,00%
Hémorragie intra-kystique	3	9,10%
Grossesse extra-utérine	9	27,30%
Volumineux kyste ovarien	5	15,20%
Appendicite aigue	1	3,00%
Non précisé	4	12,10%

Les diagnostics les plus souvent posé initialement étaient les grossesses extra-utérines et les torsions d’annexes

**Trouvailles peropératoires:** les trouvailles au cours de la chirurgie sont répertoriées dans le Tableau 4 Les trouvailles peropératoires étaient une rupture de kyste ovarien dans 36,40% des cas.

**Tableau 4:** distribution des patientes selon les trouvailles au cours de la chirurgie

Trouvailles opératoires	Effectif	Pourcentage
Torsion d'annexe	9	27,30%
hémorragie intra kystique	5	15,20%
volumineux kyste ovarien	7	21,20%
Rupture de kyste ovarien	12	36,40%

Les trouvailles peropératoires étaient une rupture de kyste ovarien dans 36,40% des cas

**Geste chirurgical pose en fonction du type de complication:** les TOPB compliquées étaient opérées en urgence dans 78,8% et par laparotomie dans 100% des cas. Le Tableau 5 montre le geste chirurgical dont a bénéficié chacune de nos patientes Le traitement était conservateur chez 26 (78,78%) patientes. Cependant, l'annexectomie a été réalisée chez 5 patientes sur 9 qui ont eu une torsion d'annexes.

**Tableau 5:** Distribution des patientes selon le geste chirurgical

Geste chirurgical	Effectif	pourcentage
Torsion d'annexe	9	
Kystectomie	3	33,30%
Annexectomie	5	55,60%
variectomie	1	11,10%
Rupture de kyste ovarien 12		
Ovariectomie	2	16,70%
Suture de la brèche	7	58,30%
Kystectomie	3	25%
Hémorragie intrakystique 5		
Kystectomie	3	60,00%
Ovariectomie	2	40,00%
Volumineux kyste ovarien	**7**	
Kystectomie	5	71,42%
Annexectomie	2	28,58%

Le traitement était conservateur chez 26 (78,78%) patientes. Cependant, l’annexectomie a été réalisée chez 5 patientes sur 9 qui ont eu une torsion d’annexes

## Discussion

Facteurs limitants: nous n'avons pas retrouvé certains registres des comptes rendus opératoires pendant la période de l'étude. Certains dossiers étaient inexploitables. Nous avons recensé 33 cas de tumeurs ovariennes compliquées ayant été opérés sur 5 ans de 2010 à 2014 dans le service de gynécologie et obstétrique de l'Hôpital Central de Yaoundé. L'âge moyen des patientes étaient de 29,33 avec un âge minimum de 20 ans et un âge maximum de 48 ans. Les tranches d'âge les plus représentées étaient 21-25 ans et de 26-30 ans soit 37,5% et 25% respectivement. Kassa a trouvé des résultats semblables avec une fréquence de 46% de TOPB dans la tranche d'âge de 20-30 ans dans sa série. Leur population d'étude était constitué en majorité de célibataire soit 55,5% ce qui concorde avec nos trouvailles où 24 (72,7%) de nos patientes étaient célibataires [4]. Ces trouvailles sont contraires à celles de Magassa au mali qui avait une population constituée de 90,8% de mariées [3]. Les multipares étaient les plus représentées dans notre série avec 45,5%. Dans notre série 33,3% de nos patientes étaient enceintes et parmi lesquels 72,7% au premier trimestre de la grossesse. Nous avons retrouvé 9 (27,27%) cas de torsion d'annexes, 12 (36,36%) cas de rupture de kyste ovarien, 5 (15,15) cas d'hémorragie intrakystique, 7 (21,21%) cas de volumineux kystes ovariens et un kyste ovarien infecté. Kassa et al ont trouvé dans une série de 68 cas de kystes ovariens, 25 cas (37%) de torsion, 20 cas (29%) d'hémorragie intrakystique, 14 cas (21%) de rupture, 7 cas (10%) de compression d´un organe voisin [4].

### Caractéristiques cliniques et thérapeutiques des complications des TOPB Torsion d'annexe

La grossesse était associée à la torsion dans 55.5% des cas. Bouguizane et al ont trouvé un taux de 17% [5]. La torsion est survenue dans 80% au premier trimestre concordant avec les trouvailles de la littérature Chang et al ayant trouvé un taux de 70% de torsion au premier trimestre de la grossesse [6]. Hasson et collaborateurs ont aussi trouvé 82% de torsion d'annexe au premier trimestre [7]. Pour ce qui est de la présentation clinique toutes nos patientes avaient une douleur pelvienne d'intensité modérée à sévère accompagnée de vomissement dans 77.8% des cas. Bar-on et al avaient trouvé dans leur étude 96% de douleur spontanée, 14% de défense localisée et 44% de vomissements [8]. Chang et al ont trouvé 95% de douleurs pelviennes, 95% de masse pelvienne, des nausées et vomissements dans 65%, la fièvre dans 10%, mais aussi une hyperleucocytose dans 45% des cas ce qui n'a pas été retrouvé dans notre série [6]. Shadinger et al dans sa série avaient retrouvé des douleurs pelviennes dans tous les cas, 85% des patientes avaient des vomissements, 56% une hyperleucocytose et 18% une fièvre, et tous ces patientes avaient des masses pelviennes [9]. Le traitement était non conservateur dans 55,6% des cas, ce taux est très élevé par rapport à celui retrouvé par Bouguizane et al qui avaient trouvé 57% de traitement conservateur [5]. Ceci peut s'expliquer par le fait que les patientes sont vues tard dans notre contexte à faible ressources. Spinelli et al avaient trouvé dans leur série 46,7% de traitement conservateur [10]. Lucas Minig et al ont trouvé que la torsion était l'indication chirurgicale dans 30,7% des cas ; ils ont réalisé l'ophorectomie dans 9 cas sur 10 [11]. Edermoglu avaient trouvé un taux de salpingo-ophorectomie à 70% [12]. Melissa et al ont aussi trouvé que le traitement était conservateur dans 30,8% des cas [13]. Nous avons retrouvé une complication à type d'avortement précoce à 9 semaines 5 jours de grossesse. Hasson et al avaient eu 2 cas d'avortement dans leur série [7]. Lucas Minig et al n'ont pas eu d'avortement, toutes les femmes ont accouché à terme [11]. Cette différence s'explique par le fait que nous avons réalisé la laparotomie pour tous nos cas tandis que Lucas Minig ont réalisé la laparoscopie qui a l'avantage d'une moindre manipulation utérine.

### Rupture de kyste ovarien

Les ruptures de kystes ovariens représentaient 36,4% des cas de notre série. Selon Toret-Labeeuw les ruptures de kyste représentent environ 3% de trouvailles coelioscopiques indiquées pour douleurs pelviennes aigues [14]. Ho et al ont trouvé une prévalence élevée chez les femmes de moins de 29ans, ce qui est en accord avec nos trouvailles car 63,6% de nos patientes avaient moins de 30ans [15]. La grossesse était associée dans notre série dans 41.70% des cas et dans 80% c'était au premier trimestre. Ce taux est assez élevé dans notre contexte car dans la littérature cette association se retrouvait dans 3 -11% des cas [16, 17]. Cliniquement toutes les patientes de notre série se sont présentées avec des douleurs pelviennes, les vomissements étaient associés dans 25% des cas, 100% avaient une sensibilité abdominale. Ces trouvailles rejoignent celles Ho et al qui trouvaient une douleur pelvienne dans 94-100% des cas, une défense dans 84% des cas et des nausées et vomissements chez 6-22% des patientes [15]. Sur le plan paraclinique la moitié de nos patientes a réalisé une numération formule sanguine et dans 66.7%, il y avait une anémie et dans 83.3% une hyperleucocytose. Ces trouvailles ont été décrites par Mishra [18]. Le diagnostic initial dans notre série était dans 50% la grossesse extra-utérine et dans 25% une hémorragie intra kystique. Les facteurs de risque comme les antécédents de rapports sexuels avant le début de la douleur, les troubles de crasse sanguine n'ont pas été évalué dans notre étude comme décrit par plusieurs auteurs Ho et al, Baidya et al, Ghafri et al [15, 19, 20]. L'échographie a pu être faite dans 75% et a retrouvé une masse annexielle dans 88.9% des cas. Un épanchement intra-abdominal a été retrouvé à l'échographie dans 44.4% des cas ce qui a également été retrouvé dans la littérature (46%.) ho et al ont trouvé que dans 65,9% le côté droit était le plus atteint, Fong et al ont montré 57,4% d'atteinte à droite ce qui est en accord avec nos résultats qui montrent une atteinte à droite dans 75% des cas [21]. Deffieux et al ont décrit la possibilité d'un traitement médical avec une surveillance rapprochée et l'administration d'acide tranxénamique [16]. Dans notre série nous n'avons recruté que les patientes ayant été opérées ce qui ne nous a pas permis d'évaluer cette option thérapeutique. Néanmoins étant donné que le diagnostic initial était la grossesse extra utérine rompue on peut comprendre pourquoi ces patientes avaient très souvent bénéficié d'un traitement chirurgical. Le traitement chirurgical était conservateur dans 83.3% des cas 7 patientes ayant bénéficié de suture de la brèche. Nous n'avons pas noté de complication post opératoire. La laparotomie était la principale voie d'abord contrairement à ho et al qui avaient 81,3% de patientes traitées par cœlioscopie [15]. Fong et al ont trouvé 78,7% de traitement par laparoscopie [21]. Mais c'est l'absence d'équipement en matériel de cœlioscopie à l'hôpital central qui justifie ce résultat

### Volumineux kystes ovariens

Nous avons trouvé 7 cas de volumineux kystes ovariens dans notre série. Les motifs de consultation fréquents étaient la douleur et la sensation de masse et la kystectomie était le geste le plus réalisé nous n'avons pas eu des signes d'obstructions des organes de voisinage.

### Hémorragie intrakystique

5 cas d'hémorragie intrakystique ont aussi été répertoriés. Il s'agissait de trouvailles opératoires alors que le diagnostic initial n'était pas un kyste ovarien hémorragique. Le motif de consultation était la douleur dans 100% des cas et la kystectomie a été faite dans 60% des cas. Deffieux et al pensent que les signes cliniques d'hémorragie intrakystique ne sont pas spécifiques [16].

## Conclusion

Les tumeurs ovariennes présumées bénignes compliquées sont présentes dans notre milieu surtout chez des patientes en âge de procréer. La présentation clinique varie en fonction du type de complications. Le diagnostic tardif particulièrement en cas de torsion d'annexe oblige un traitement radical qui est délétère pour le patient. Le traitement chirurgical par laparoscopie est le plus recommandé si le plateau technique le permet car il diminue les risques de complications.

### Etat des connaissances actuelle sur le sujet

- Environ 5% des femmes développent un kyste de l'ovaire au cours de leur vie, ils peuvent survenir à tout âge;
- Les kystes ovariens sont bénins dans 95% des cas;
- Leur incidence pendant la grossesse est de 0,2 à 5%.

### Contribution de notre étude à la connaissance

- La douleur est le maître symptôme des complications des tumeurs ovariennes présumées bénignes;
- La laparotomie était pratiquée chez toutes les patientes à cause du faible plateau technique contrairement dans la littérature où le traitement est essentiellement par laparoscopie.

## References

[1] Kaouther D, Hajeur B, Mohammed D, Amel T, Mohammed FG (2014). Kystes de l'ovaire: score échographique de malignité. Pan Afr Med J..

[2] Mimoun C, Fritel X, Fauconnier A, Deffieux X, Dumont A, Huchon C (2013). Epidémiologie des tumeurs ovariennes présumées bénignes. Journal de gynécologie obstétrique et biologie de la reproduction..

[3] Magassa RK Aspects clinique et épidémiologique et prise en charge des kystes ovariens au CSREF-C VI..

[4] Kassa A Aspects épidémiologiques de kyste de l'ovaire. Cas l'hôpital général de référence Charité maternelle de Goma, de 01/01/2008 au 31/12/2011..

[5] Bouguizane S, Bibi H, Farhat Y, Dhifallah S, Darraji F, Hidar S (2003). Adnexal torsion: a report of 135 cases. J Gynecol Obstet Biol Reprod..

[6] Chang SD, Yen CF, Lo LM, Lee CL, Liang CC (2011). Surgical intervention for maternal ovarian torsion in pregnancy. Taiwan J Obstet Gynecol..

[7] Hasson J, Tsafrir Z, Azem F, Bar-On S, Almog B, Mashiach R (2010). Comparison of adnexal torsion between pregnant and non-pregnant women. Am J Obstet Gynecol..

[8] Bar-On S, Mashiah R, Stockheim D, Soriano D, Goldenberg M, Schiff E (2010). Emergency laparoscopy for suspected ovarian torsion: are we too hasty to operate?. Fertil Steril..

[9] Shadinger LL, Andreotti RF, Kurian RL (2008). Preoperative sonographic and clinical characteristics as predictors of ovarian torsion. Journal of Ultrasound in Medicine..

[10] Spinelli C, Buti I, Pucci V, Liserre J, Alberti E, Nencini L (2013). Adnexal torsion in children and adolescents: new trends to conservative surgical approach - Our experience and review of literature. Gynecological Endocrinology..

[11] Lucas M, Lucas O, Pilar C, María Guadalupe P, Cecilia B, Ignacio Z (2016). Laparoscopic surgery for treating adnexal masses during the first trimester of pregnancy. J Minim Access Surg..

[12] Erdemoglu M, Kuyumcuoglu U, Kale A (2010). Pregnancy and adnexal torsion: analysis of 20 cases. Clin Exp Obstet Gynecol..

[13] Melissa W, Julian S (2005). Ovarian torsion: 10-year perspective. EMA..

[14] Toret-Labeeuw F, Huchon C, Popowski T, Chantry AA, Alexandre Dumont A, Fauconnier A (2013). Routine ultrasound examination by OB/GYN residents increase the accuracy of diagnosis for emergency surgery in gynecology. World Journal of Emergency Surgery..

[15] Ho WK, Wang YF, Wu HH, Tsai HD, Chen TH, Tsai H (2009). Ruptured corpus luteum with hemoperitoneum: case characteristics and demographic changes over time. Taiwan J Obstet Gynecol..

[16] Deffieux X, Thubert T, Huchon C, Demoulin G, Rivain AL, Faivre E (2013). Complications de tumeurs ovariennes présumées bénignes. Journal de gynécologie obstétrique et de biologie de la reproduction..

[17] Ajanli A, Poonam G, madhori W, Rimpy M, Sarla M (2004). Ruptured corpus luteum with hemoperitoneum. Journ Obstet ind..

[18] Mishra J (2013). Accidents to ovarian cyst. Journal of universal college of medical sciences..

[19] Ghafri WA, Gowri V, Khaduri MA, Shukri MA (2013). Life threatening corpus luteal hemorrhage. Gynecology..

[20] Baidya JL, Chakraborty J, Ray J, Pradhan M (2015). Thrombocytopenia & Ruptured Corpus Luteal CYST, a Deadly Combination: a Case Report. Journal of Evolution of Medical and Dental Sciences..

[21] Fong YF, Hui, Chua HW, Singh C, Tan SH (2013). Diagnostic Dilemma: acute abdomen from ruptured corpus luteum requiring surgical intervention in young women. Thai Journal of Obstetrics and Gynaecology..

